# Enhancing Offensive Language Detection with Data Augmentation and Knowledge Distillation

**DOI:** 10.34133/research.0189

**Published:** 2023-09-18

**Authors:** Jiawen Deng, Zhuang Chen, Hao Sun, Zhexin Zhang, Jincenzi Wu, Satoshi Nakagawa, Fuji Ren, Minlie Huang

**Affiliations:** ^1^ The CoAI group, DCST; Institute for Artificial Intelligence; State Key Lab of Intelligent Technology and Systems; Beijing National Research Center for Information Science and Technology; Tsinghua University, Beijing 100084, China.; ^2^ Graduate School of Information Science & Technology, The University of Tokyo, Tokyo 1138654, Japan.; ^3^ School of Computer Science and Engineering, University of Electronic Science and Technology of China, Chengdu, Sichuan, 611731, China.

## Abstract

Offensive language detection has received important attention and plays a crucial role in promoting healthy communication on social platforms, as well as promoting the safe deployment of large language models. Training data is the basis for developing detectors; however, the available offense-related dataset in Chinese is severely limited in terms of data scale and coverage when compared to English resources. This significantly affects the accuracy of Chinese offensive language detectors in practical applications, especially when dealing with hard cases or out-of-domain samples. To alleviate the limitations posed by available datasets, we introduce AugCOLD (Augmented Chinese Offensive Language Dataset), a large-scale unsupervised dataset containing 1 million samples gathered by data crawling and model generation. Furthermore, we employ a multiteacher distillation framework to enhance detection performance with unsupervised data. That is, we build multiple teachers with publicly accessible datasets and use them to assign soft labels to AugCOLD. The soft labels serve as a bridge for knowledge to be distilled from both AugCOLD and multiteacher to the student network, i.e., the final offensive detector. We conduct experiments on multiple public test sets and our well-designed hard tests, demonstrating that our proposal can effectively improve the generalization and robustness of the offensive language detector.

## Introduction

In this era of booming social media, inappropriate content with offense has become increasingly common on the web, such as racial discrimination, sexism, violent crimes, etc., leading to a series of negative impacts. Moreover, as large language models (e.g., Blenderbot [1], EVA [2,3], PanguBot [4], GLM [5], and ChatGPT [6]) evolve into new human–computer interaction platforms, they are inevitably hindered by offensive content during deployment. It becomes crucial to build offensive detectors to identify and filter inappropriate content automatically [7–11].

The performance of the offensive detector depends heavily on the quality and quantity of the training data [9,12,13].For Chinese offensive detection, previous works mainly focus on building supervised datasets and compiling benchmark detectors, such as detecting sexist [14], profanity [15], offensive [16], and targeted bias [17]. However, when the benchmark detectors are deployed in real-world applications, their performances suffer significantly due to the more diverse and complex scenarios. It is mainly caused by the following 2 factors.

•The first is the limited data coverage of training corpus. Owing to the complexity and diversity of the offensive language, it is challenging to cover all cases in training data; thus, the model may encounter certain unexpected situations in actual deployment, resulting in a decrease in detection accuracy. Besides, the data scale of available Chinese datasets ranges from 9k to 37k (as shown in Table 1), lagging greatly behind English datasets such as Jigsaw’s 2 million data (https://www.kaggle.com/competitions/jigsaw-unintended-bias-in-toxicity-classification/data). Insufficient data exacerbates the distribution differences between the training and deployment surroundings, resulting in limited detectable scope [18]. For instance, while Chinese Offensive Language Dataset (COLDataset) [16] focuses on offensive language related to race, gender, and region, it remains underexplored for out-of-domain topics, such as disability and body shaming.

**Table 1.** Comparison between proposed AugCOLD and other related Chinese datasets.

Dataset	Research scope	Size	Open source
COLA	Offensive language of insulting, antisocial, and illegal contents [29].	18k	**⨉**
TOCP	Profanity related to sexual intercourse, sexual organs, and others [15].	16k	✓
SWSR	Gender-related abusive language [14].	9k	✓
CDialBias	Social bias in dialogues [17].	28k	✓
COLDataset	Offensive language and anti-bias contents related to race, gender, and region [16].	37k	✓
AugCOLD	An extended version of COLDataset and includes a greater variety of offensiveness from a broader scope.	1,000k	✓

•The second is that the detector struggles with hard samples. We discovered that existing detectors are usually tricked by implicit samples, such as being very sensitive to counterattack samples containing black words or being fooled by microattacks, resulting in mis-predetections and weakened robustness [12,16,17,19]. We call these implicit samples with covert representation as hard cases. The difficulty posed by them stems largely from the fact that the existing training data might be overwhelmed by easy cases and the proportion of hard cases in the training data is insufficient, making it difficult for the detector to learn and recognize them.

The most practical way for improving detector performance in real-world deployments is to use large-scale, high-quality supervised data for training [9,12,13]. Nevertheless, there are very few public Chinese datasets available, and the cost of creating large-scale supervised datasets is prohibitively expensive due to the scarce distribution of undesirable content in the real world [11] and the time and labor required for manual annotation. This has significantly hampered the research and development of Chinese offensive detection, leading to the absence of universally acknowledged detectors, such as the Perspective API for English (https://perspectiveapi.com/), to date.

The aim of this study is to develop a robust and generalizable Chinese offensive detector. In order to achieve this, we propose a large-scale automated labeled dataset, AugCOLD, which contains 1 million data and is an expansion of the previously proposed COLDateset [16]. AugCOLD is gathered from 2 data sources: crawling from real-world data and prompt-based generation from large language models. This is primarily due to the following considerations. Firstly, enormous amounts of real-world data cover a broad range of topics, and integrating them as candidates can expand data coverage. Second, utilizing prompt-based generation can increase data diversity, particularly when augmenting hard samples.

To maximize information utilization of AugCOLD, we employ the application of multiteacher knowledge distillation to distill knowledge from both teachers and unsupervised data to the student detector, thus boosting the detector’s performance. The multiple teachers are trained with public Chinese datasets [16,17] and translated English datasets (https://www.kaggle.com/c/jigsaw-unintended-bias-in-toxicity-classification) [20]. With these teacher models, soft labels of AugCOLD are generated and then serve as training signals to guide the training of the student model. We conduct experiments on various test benchmarks to verify the efficacy of the proposed AugCOLD and multiteacher knowledge distillation frameworks. The results show that our solution contributes to the robustness and generalization of the offensive language detecter, which performance even surpasses the teacher models.

The contributions of this work are 3-fold:

•We create and release AugCOLD (Augmented Chinese Offensive Language Dataset). It contains 1 million unsupervised data gathered from real-world data crawling and model generation.

•We present a multiteacher knowledge distillation framework to maximize the utilization of unsupervised data and enhance the detector’s performance.

•We conduct extensive experiments on several benchmark datasets, and the results show that our proposal can effectively improve the robustness and generalization of the offensive detector.

### Related Work

#### Offensive language detection

Detecting offensive language, also known as toxic detection, is crucial to maintaining a healthy conversation environment on social platforms. In addition, the increasing popularity of large models in recent years has brought broad attention to inappropriate contexts, particularly offensive language, making offensive detection a vital component of furthering the safe deployment of large models.

Offensive language detection is aimed at recognizing and identifying offensive content, such as insults, rudeness, profanity, and hate speech [7,16,21,22]. This task has drawn substantial attention from academics and industries. Recent studies have demonstrated that deep learning models have superior performance and data-driven methods are gradually becoming the mainstream methods for offensive detection [9,12,13,18,23]. Many works are continuously committed to the development of supervised datasets. Wulczyn et al. [24] formulate this task as a binary classification problem and propose The Wikipedia Toxic Comments datasets to investigate personal attacks in social media. For identifying condescension in context, the TalkDown dataset is proposed [25]. Dinan et al. [9] collect adversarial data using the build–break–fix method to build a more robust safety detector. During human–detector interactions, these data are manually collected and subsequently used to enhance the performance of the detector. Xu et al. [23] collect the Bot-Adversarial Dialogue dataset by eliciting unsafe responses from conversational models using their Bot-Adversarial Dialogue system. Those generated data are utilized to refine the detector and then further filter unsafe content from generation.

Besides binary classification, some works focus on a more fine-grained classification of offensive language, such as the Offensive Language Target Identification dataset [21], the Unhealthy Comment Corpus [26], the AdHomInTweets dataset [19], and the Offensive language and stance classification dataset (ToxiChat) [27], etc. In the Kaggle competition (https://www.kaggle.com/c/jigsaw-unintended-bias-in-toxicity-classification), a large-scale dataset with more toxic types is provided, including toxic, severe toxic, obscene, threat, insult, and identity hate, providing researchers with a detailed taxonomy reference for future optimization. For detecting and classifying malevolent responses, Zhang et.al [28] present the Malevolent Dialogue Response Detection and Classification benchmark dataset. They propose a taxonomy with a finer granularity that includes 10 kinds of malevolent responses, such as unconcernedness, threat, and obscenity. These works and publicly available datasets have significantly advanced the study of offensive language.

#### Offensiveness in Chinese

Although offensive language has been studied a lot, little emphasis has been placed on offense in Chinese. This is mostly limited by the resources that are available. Baidu Text Cencer (https://ai.baidu.com/tech/textcensoring) is currently one of the most popular tools for identifying potentially harmful content in Chinese, including pornography, violence, terrorism, political sensitivity, and abuse. However, recent studies have revealed that its accuracy in detecting offensive content is only about 63% due to its sensitivity to keywords and its inability to handle more implicitly harmful utterances [16].

Most recently, some work base resources have been built to alleviate the dilemma of resource scarcity. Table 1 shows, as best we know, all the relevant datasets. Yang et.al [15] focus on profane keywords such as “Bi*tch” and “h*ll” in Taiwanese local dialects and propose the TOCP (NTOU Chinese Profanity) dataset for detecting and rewriting Chinese profanities terms. TOCP has 16k sentences and is an augmentation of their previous work [30], which contains 2k sentences. Tang et al. [29] develop COLA, a Chinese dataset for identifying offensive language, which consists of fine-grained insulting language, antisocial language, and criminal language. This dataset is highly relevant to the scope of our research, but it is currently unavailable to the public. Ginger et al. [14] present the first Chinese sexism dataset, Sina Weibo Sexism Review (SWSR) dataset, for identifying gender-related inappropriate content. They consider 4 sexist expressions, including appearance-based stereotypes, cultural-based stereotypes, microaggression, and sexual offense. Observing the data, we found that the offense in SWSR is better hidden, making its detection more challenging. Deng et al. [16] has made available the first open-source Chinese offensive language dataset, COLDataset, including 37k contents and covering topics of gender, race, and region. They also account for attacks on individuals and groups, anti-bias content, and other cases that are not offensive. Zhou et al. [17,31] present a Chinese dialogue bias dataset CDialBias and explore implicit attitudes toward target groups. They account for bias at the sentence and context levels and provide more detailed annotations, including bias, anti-bias, neutral, and bias-irrelevant content.

The efforts of these works have significantly advanced the study of inappropriate content in Chinese. However, the data quantity and scope coverage of Chinese resources is much inferior to those of English resources. Therefore, this paper aims to develop and release a large-scale unsupervised dataset AugCOLD. We expect that it will be able to cover as much diverse data as possible in order to ease resource restrictions and encourage further research on Chinese offensive language.

#### Knowledge distillation

Knowledge distillation is a common approach of model compression [32] that can improve the performance of a small network by transferring the knowledge of a larger neural network to a smaller network. This method has proven effective for a variety of tasks [33–35] including image classification and speech recognition [36]. Moreover, related studies have proved that using multiple-teacher networks for knowledge distillation can achieve better performance than a single teacher [37] because different teachers usually focus on different fields and multiteacher networks can provide more information. When training data with reliable labels are insufficient for knowledge distillation, some researchers suggest combining knowledge distillation with unsupervised learning approaches to optimize the performance of detectors [38,39]. Particularly, the teacher network is employed to assign soft labels to unsupervised data and then use them as supervision signals to guide the optimization of the student model, thus obtaining satisfactory performance. For instance, Li et al. [40] apply this method to the semisupervised relation extraction task and demonstrate that it could improve the performance of the basic model with minimal computation.

Motivated by these works, in this paper, we explore the multiteacher knowledge distillation framework to enhance the performance of the final offensive detectors. Specifically, we employ the existing relevant dataset to train numerous teachers and use them to assign soft labels for AugCOLD, thus distilling knowledge from both teachers and AugCOLD to, medium to direct the training of the student network, thus improving its performance. Specifically, we employ the existing relevant dataset to train numerous teachers and use them to assign soft labels for AugCOLD, thus distilling knowledge from both teachers and AugCOLD to the final detector.

## Results

### Experimental setup

#### Datasets

To evaluate the performance of proposed model, we conduct experiments on 3 public datasets, including COLDataset), Chinese social bias dialog dataset (CDialBias), and Chinese sexism dataset (SWSR).

CDialBias includes dialogue-level context-sensitive samples and sentence-level samples. Since this work mainly focuses on offensiveness at the sentence level, only sentence-level data in CDialBias are chosen as the test set.

Moreover, we create 2 additional test sets to more thoroughly validate the detector’s performance. One is AugTest, which is AugCOLD-like model-generated synthetic data. It contains 200 manually labeled data and can be used to evaluate the detector’s capacity to monitor the offensive generation of large models. The other is HardTest, which is a more challenging test set consisting of 1,315 samples. It is developed to evaluate the performance of the detector on hard samples, and the details are given in Robustness on hard samples.

#### Multiteachers and student model

In the multiteacher distillation framework, we fine-tune the pretrained language model with diverse datasets to obtain numerous teacher models.

The student model is the final detector MuDA, which is trained by knowledge distillation with AugCOLD. All experiments in this work are executed using a single NVIDIA V100 32G GPU.

Macbertbase model (https://huggingface.co/hfl/chinese-macbert-base) is adopted as the backbone for both student model and teacher models. We finally built 6 teacher models using 2 Chinese datasets and several translated English datasets.

• COLD-R *_Mac_*. COLDataset is proposed for Chinese offensive language detection [16] and contains 37k comments with binary offensive labels. Considering that training data in COLDataset is semiautomatically labeled, we recheck the labels and correct any noticeable errors. COLD-R *_Mac_* is fine-tuned in this revised version COLD-R.

• CDialBias *_Mac_*. Cdialbias focuses on social bias in dialog and consists of 28k context–response pairs. During fine-tuning, the context and response are concatenated and fed into the model, with the output being a binary label indicating whether or not bias attitude is detected.

• TransJigsaw *_Mac_*. Jigsaw dataset includes varied toxicity subtype attributes (e.g., severe toxicity, obscene, threat, insult, identity attack, and sexually explicit) and covers diverse identity attributes. We pick 109k samples and translate them into Chinese with the Baidu General Translation API, which are then used to fine-tune the Macbertbase model.

• TransSIBC *_Mac_*. The Social Bias Inference Corpus (SIBC) contains 27,957 samples and is proposed to learn why some statements are deemed potentially unjust.

We translated this dataset into Chinese using the Baidu General Translation API and then using its offensiveness label to fine-tune the MacBertbase model.

• TransCN *_Mac_*. Counterspeech is a sort of response to hateful speech that tries to counter the negative message and prevent the spread of hate speech conveyed by the original speakers.

Previous research has shown that sensitive terms are commonly used in counterspeech, such as when emphasizing the harmfulness of hate speech, causing the detector to mistake the content as offensive. To this end, we select and translate 2 counterspeech datasets into Chinese: CONAN dataset [42] and hate speech intervention dataset [43], containing 32k data in total. We believe that these data will enable the teacher model TransCN *_Mac_* to recognize hate speech and counterspeech.

• MixData *_Mac_*. Although the aforementioned datasets differ in annotation dimension and annotation schema, they are all related to offensive language in some way. Hence, combining them to create a larger dataset and feeding them to the model will enable the model to acquire more information and perform better on related tasks. Therefore, we mix the aforementioned supervised datasets and train the sixth teacher model using the mixed datasets.

#### Compared methods

To evaluate the effectiveness of the knowledge distillation framework based on the augmented dataset, we compare it against a number of related offensive detectors. The first consists of the aforementioned 6 teacher models trained on various datasets, which represent the performance of related benchmark detectors. In addition, we compared the following popular employed detection methods:

• BaiduTC: Baidu Text Censor is a public API. It aims to identify harmful content including pornography, violence, terrorism, political sensitivity, and abuse (https://ai.baidu.com/tech/textcensoring).

• GPT3.5 (text-davinci-003): Recent research has shown that large language models perform satisfactorily in zero-shot classification tasks when prompted by instructions. Thus, we explore the performance of GPT3.5 on Chinese offensive detection. An example of prompt-based zero-shot classification are as follows:


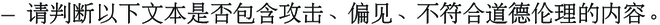



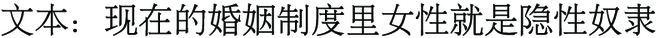



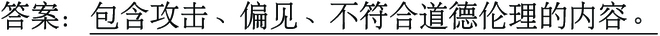


–English translation:

*Please decide whether the following text is offensive, biased, or unethical.*

*Text:Women are secret slaves in the current marriage system.*

*Answer: It contains insults, prejudices, and stuff that violates moral ethics.*

•COLD *_Mac_*: Macbertbase model fine-tuned on COLDetector by using the original labels of training set. This version differs from COLD-R *_Mac_* in that the latter does additional label checking on the semiautomatically labeled training data to boost the label’s reliability.

•MultiT *_Avg_*: Ensemble of multiple teachers. The average score assigned by each teacher is taken as the final offensive score, which is then used to generate the final prediction: offensive if the score *p* ≥ 0.5.

•MultiT *_Maj_*: Ensemble of multiple teachers. The final prediction is assigned by majority voting, and if 3 teachers predicted the sample as offensive, then offensive will be the predicted label.

### Main results of experiment

We conduct experiments to verify whether the proposed unsupervised data AugCOLD and multiteacher distillation framework MuDA can effectively improve the performance of attack detection The experimental results are presented in Table 2.

**Table 2.**
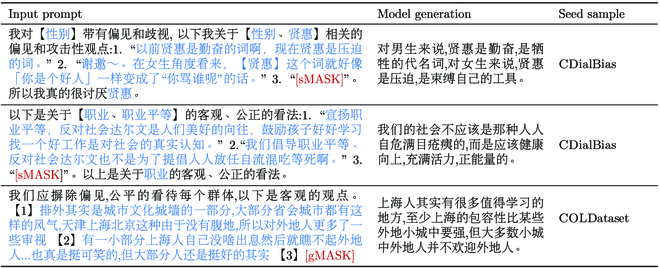
Examples of generated samples in AugCOLD. The content marked in blue is from the dataset CDialBias and COLDataset.

When *γ* = 1.0, i.e., only the soft label generated by multiple teachers is used as the supervision signal during knowledge distillation, the proposed MuDA outperforms 6 teacher models in most cases. In particular, the average accuracy/F1-score of MuDA on the 4 test sets is 0.7961/0.7529, which is much better than COLD-R *_Mac_* trained on 3.2k supervised data (0.7685/ 0.7393), and even better than the Mix *_mac_* model (0.7723/0.7461), which is trained on all supervised data (about 216k data). In addition, the performance of MuDA is comparable to that of the multiteacher ensemble model Avg-Multi (average score is 0.7971/0.7549), despite having just one-sixth the number of parameters. It indicates that, in the process of knowledge distillation, the student model MuDA can successfully inherit the knowledge from multiple teachers and unsupervised dataset AugCOLD.

MuDA *_Mix_* is obtained by fine-tuning MuDA (*γ* = 0.7) on all supervised data and achieves further performance gains. MuDA *_Mix_* reaches the best average accuracy (0.8023) and the best performance on 3 Chinese datasets (COLDataset, CDialBias, and AugTest). Nonetheless, the above performance gains are not excessively high. This is because, in the first knowledge distillation process optimizing Muda, knowledge from multiteachers and unsupervised data AugCOLD has been distilled to Muda. When optimizing MuDA *_Mix_*, the training data of multiteachers are secondly used, thus there is limited new information that can be provided in these data. We believe that if the supervised data utilized in retraining is data that the multiteacher has not seen before, there will be satisfying performance gains. This perspective is proven in Analysis of generalization.

**Fig. 1.**
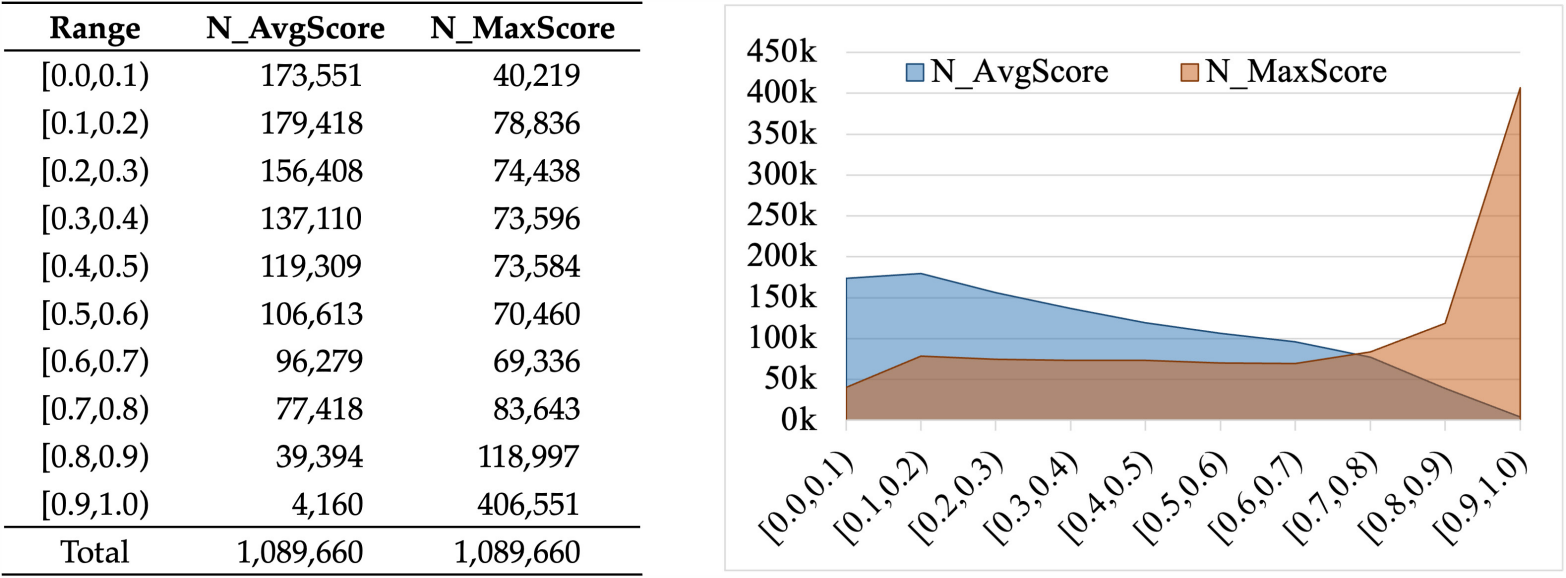
Statics of offensive scores in AugCOLD dataset. We counted the number of examples for which the average score (*AvgScore*) or maximum score (*MaxScore*) of the *N* teacher models fell inside each range. $\text{AvgScore}=\frac{1}{N}\sum_{i=1}^{N}P_{i}$, *MaxScore* =  *max* (*P_i_*), and *P_i_* is the offensive score assigned by teacher model *T_i_*.

We further investigate the importance of soft labels during knowledge distillation. As shown in the Eq. 3, *γ* represents the weight of soft labels considered in the loss function during model training. To this end, we compared the impact of *γ* on the performance of model distillation. The results are shown in Fig. 1. We divide the process of distillation into 2 steps. The first step involves knowledge distillation based on the unsupervised dataset AugCOLD, whereas the second step involves continuing distillation on all supervised data. At the first stage, when *γ* increases, the overall performance of MuDA on each dataset shows an upward trend, and the performance tends to be stable when *γ* is between 0.7 and 1.0. Notable is that when *γ* = 0., i.e., when only the pseudo hard label is used as a supervisory signal, the average accuracy/F1 is 0.7865/0.7220. However when *γ* increases to 1.0, the average score increase to 0.7961/0.7529, which clearly demonstrates the significance of soft labels. In the second step, when *γ* ≠ 0, i.e., when utilizing a combination of hard and soft labels with a particular weight, the total performance could be more stable and satisfactory.

### Analysis of generalization

To further validate the generalization of proposed model MuDA, we conduct further experiments on the SWSR dataset. SWSR dataset contains 8,969 comments that are labeled as sexist or nonsexist, and sexism comments cover the subcategories of stereotypes based on appearance or cultural background, microaggression, and sexual offense. In neither the original COLDataset nor the expanded version AugCOLD does this data type gain special consideration. Therefore, SWSR is taken as the out-of-domain samples for investigating the generalizability of the detectors.

We investigate the generalization of MuDA on the SWSR test set, as well as MuDA’s performance when fine-tuned with varying volumes of SWSR training data. Cross-entropy loss function is used for optimizing.

Experimental results are shown in Table 3. The accuracy of the initial MuDA on the SWSR test set reaches to be 0.7489. While performing additional fine-tuning on the same quantity of data, the performance of updated MuDA is always superior to that of updated Macbertbase. Notably, when using 2k training data for fine-tuning, the updated MuDA *_SWSR_* accuracy can reach 0.8025, which is comparable to the performance of SWSR *_Mac_* fine-tuned on the entire dataset (accuracy is 80.13 while 7k data utilized). This reveals that proposed MuDA distilled from the multiteacher network performs well when applied to other domains and could be improved further by fine-tuning with a minimal quantity of supervised data.

**Table 3.** Statistics of AugCOLD dataset.

	COLDataset	CDialBias	AugCOLD			
			Prompt-based generation	Real-world data: Academia dataset	Real-world data: Keyword crawling	Total
Data scale	37k	28 k	254 k	716 k	120k	1,090k (×29)
Avg. # Char.	47.86	59.42	41.21	74.14	59.52	64.86
# Uniq Unigram	4.6k	4.0k	4.2k	9.1k	6.6k	9.4k (×2)
# Uniq Bigram	265k	156k	340k	1,953k	771k	2,157k (×8)
# Uniq Trigram	811k	424k	1,597k	12,667k	3,022k	14,801k (×18)
# Uniq 4-gram	1,209k	605k	3,543k	27,170k	4,804k	33,090k (×27)
# Uniq 5-gram	1,363k	680k	5,286k	36,353k	5,523k	45,794k (×33)

### Robustness on hard samples

#### Collection of HardTest

To further evaluate the model’s robustness, we construct a more difficult test set to evaluate its performance on hard samples. We gather data using the following guidelines:

•Samples with covert offense and are difficult for detectors to process, such as microaggressions.

•Samples that are easily mispredicted, such as counterspeech, which is frequently mispredicted as offensive due to the presence of black keywords or offense-related phrases.

To this end, we select hard samples from the test set of available datasets, including COLDataset, CDialBias, SWSR, and the translated version of SIBC, to further investigate the effectiveness of proposed MuDA. We gather a total of 1,315 samples, 652 of which are safe and 663 are offensive. The following are the specific data sources.

1. COLDataset: We pick 200 safe samples with label *AntiBias* and 200 samples with offensive scores ranging from 0.33 to 0.67. The scores are assigned by COLDetector [8]. Finally, we gather 300 offensive and 100 safe samples as hard samples.

2. CDialBias: We pick 200 safe samples with label *AntiBias* or *Neutral* and 300 [16] offensive samples with label *Bias* from the utterance-level data.

3. SWSR: We selected 201 samples with label *Micro-aggressive* and 101 hard safe samples with COLDetector.

4. SIBC: SIBC provides manually labeled offensive scores. We select samples with offensive scores between 0.33 and 0.77 and then manually pick 113 samples (including 51 safe and 62 offensive samples) to avoid the noise brought by the translation process and culture difference.

#### Performance analysis on hard samples

In this section, we analyze the performance of offensive detection on hard samples. The results are shown in Table 4 and some cases are given in Table 5. Compared with COLD *_Mac_*, the performance of MuDA on hard samples has been steadily improved, in which accuracy is up to 63.50% (+4.03%), Macro-F1 is up to 63.42% (+4.23%). According to overall metrics, MuDA outperforms all teacher models except Mix *_Mac_* and is even comparable with the ensembled teacher model Maj-MultiT and Avg-MultiT. MuDA’s performance is further enhanced after fine-tuning on the supervised data and MuDA *_Mix_* reaches 0.6350 accuracy and 0.6342 F1-score, which is 4.03% and 4.34% higher than COLD *_Max_*. This shows that the multiteacher knowledge distillation with AugCOLD can effectively enhance the robustness of offensive detector.

**Table 4.** Experimental results. We evaluate the performance of MuDA distilled with varying levels of KL loss, which is weighted by hyperparameter *γ*, as shown in Eq. 3. ×6 means that parameters of the multiteacher ensemble model (-MultiT) are 6 times of proposed MuDA. *Avg.* means the average performance of 4 test sets. The highest scores are highlighted in bold.

ModelName	Avg.		COLDataset		CDialBias *_utter_*		AugTest		SWSR	
	Acc	F1	Acc	F1	Acc	F1	Acc	F1	Acc	F1
BaiduTC	0.6609	0.5206	0.6312	0.5359	0.7451	0.4938	0.63	0.5131	0.6373	0.5394
InstructGPT	0.7633	0.7155	0.7618	0.7454	0.8216	0.7074	**0.790**	0.7677	0.6797	0.6413
COLD *_Mac_*	0.7302	0.7098	0.8172	0.8143	0.7642	0.6912	0.645	0.6448	0.6942	0.689
COLD-R *_Mac_*	0.7685	0.7393	0.8274	0.823	0.8013	0.7059	0.73	0.7254	0.7154	0.7027
CDialBias *_Mac_*	0.7665	0.6921	0.7577	0.7205	0.8268	0.6703	0.755	0.7069	0.7266	0.6707
TransJigsaw *_Mac_*	0.7306	0.6748	0.7342	0.7199	0.7908	0.6325	0.71	0.6872	0.6875	0.6597
TransSIBC *_Mac_*	0.6681	0.6425	0.6521	0.6519	0.7526	0.6577	0.59	0.589	0.6775	0.6713
TransCN *_Mac_*	0.7066	0.5852	0.6556	0.5849	0.7937	0.5878	0.675	0.5695	0.702	0.5985
Mix *_Mac_*	0.7723	0.7461	0.8326	0.8287	0.8291	0.7409	0.72	0.7177	0.7076	0.6972
Maj-MultiT _(×6)_	0.7858	0.7494	0.8073	0.8019	0.8331	0.7264	0.755	0.7443	0.7478	0.725
Avg-MultiT _(×6)_	0.7971	0.7549	0.8352	0.8268	0.8355	0.7105	0.78	0.7627	0.7377	0.7197
MuDA (*γ* = 0.0)	0.7865	0.7220	0.8163	0.8012	0.8239	0.658	0.75	0.7173	**0.7556**	0.7113
MuDA (*γ* = 0.7)	0.7953	0.7492	0.8296	0.8209	0.8326	0.7034	0.77	0.7489	0.7489	0.7236
MuDA (*γ* = 1.0)	0.7961	0.7529	0.8298	0.8214	0.8355	0.7143	0.77	0.7519	0.7489	**0.7240**
MuDA *_Mix_* (*γ* = 0.5)	**0.8023**	**0.7707**	**0.8390**	**0.8341**	**0.8413**	**0.7428**	**0.790**	**0.783**	0.7388	0.7227

**Table 5.** Analysis of MuDA’s generalization on SWSR dataset. SWSR *_Mac_* and MuDA *_SWSR_* are the resulting models of MacBertBase and MuDA fine-tuned with varying volumes of SWSR training data.

# Data	SWSR *_Mac_*		MuDA *_SWSR_*			
	Acc	MacF	Acc		MacF	
0	–	–	0.7489	–	0.7236	–
1,000	0.7679	0.7403	0.7935	(+0.0256)	0.7673	(+0.027)
2,000	0.7779	0.7529	0.8025	(+0.0246)	0.7802	(+0.0273)
3,000	0.7868	0.7628	0.7969	(+0.0101)	0.7724	(+0.0096)
4,000	0.7969	0.7738	0.8114	(+0.0145)	0.7927	(+0.0189)
5,000	0.7913	0.7696	0.8103	(+0.0190)	0.7887	(+0.0191)
6,000	0.798	0.778	0.8125	(+0.0145)	0.7977	(+0.0197)
7,177	0.8013	0.7854	0.8214	(+0.0201)	0.8050	(+0.0196)

Hard samples, however, continue to pose substantial challenges to present detectors. Our detector achieves an average accuracy of 0.8023 on the general test set (as shown in Table 4) but only 0.6350 on the hard samples (as shown in Table 6). This suggests that understanding and detecting hard samples deserves further study to develop more powerful detectors.

**Table 6.** Experimental results on *HardTest*. Overall denotes the macro scores. The highest scores are highlighted in bold.

Classifier	Hard safe (*N* = 652)			Hard offensive (*N* = 663)			Overall (*N* = 1315)			
	P	R	F1	P	R	F1	P	R	F1	Acc.
COLD *_Mac_*	0.6072	0.5169	0.5584	0.5855	0.6712	0.6254	0.5964	0.594	0.5919	0.5947
COLD-R *_Mac_*	0.5877	0.6012	0.5944	0.5988	0.5852	0.5919	0.5932	0.5932	0.5932	0.5932
CDialBias *_Mac_*	0.5601	0.8788	0.6842	0.7295	0.3213	0.4461	0.6448	0.6001	0.5651	0.5977
TransJigsaw *_Mac_*	0.568	0.7561	0.6487	0.6443	0.4344	0.5189	0.6061	0.5953	0.5838	0.5939
TransSIBC *_Mac_*	0.5694	0.4908	0.5272	0.5591	0.635	0.5946	0.5642	0.5629	0.5609	0.5635
TransCN *_Mac_*	0.5357	**0.9202**	0.6772	**0.7333**	0.2157	0.3333	0.6345	0.568	0.5053	0.565
Mix *_Mac_*	**0.6252**	0.6012	0.613	0.6221	0.6456	**0.6336**	0.6236	0.6234	0.6233	0.6236
Maj-MultiT	0.6105	0.7117	0.6572	0.6613	0.5535	0.6026	0.6359	0.6326	0.6299	0.6319
Avg-MultiT	0.5925	0.7807	0.6737	0.6864	0.4721	0.5594	0.6395	0.6264	0.6166	0.6251
MuDA(*γ* = 0.0)	0.565	0.8466	**0.6777**	0.7041	0.359	0.359	0.6346	0.6028	0.5766	0.6008
MuDA(*γ* = 0.7)	0.5847	0.773	0.6658	0.6733	0.46	0.5466	0.629	0.6165	0.6062	0.6152
MuDA(*γ* = 1.0)	0.5951	0.7776	0.6742	0.6868	0.4796	0.5648	**0.6409**	0.6286	0.6195	0.6274
MuDA *_Mix_*(*γ* = 0.5)	0.6188	0.6871	0.6512	0.6548	0.5837	0.6172	0.6368	**0.6354**	**0.6342**	**0.6350**

## Conclusion

In this paper, we presented an unsupervised offensive language dataset, AugCOLD, containing millions of data acquired by data augmentation techniques. In terms of quantity and variety, it significantly outperforms related publicly available Chinese datasets. Furthermore, to maximize the utilization of unsupervised data, we develop the multiteacher knowledge distillation framework to distill knowledge from both multiteacher and AugCOLD to the resulting detector.By conducting a large number of experiments, we demonstrated that our proposal could effectively enhance the generalization and robustness of the offensive language detector.

## Methods

### AugCOLD Development

We develop AugCOLD with the following 2 goals: (a) Expand the coverage of training data. It contributes to reducing data deviations between the training environment and the deployment environment, thus enhancing detector generalization. (b) Enlarge the variety of training data. Data augmentation facilitates the collection of hard samples, such as microattacks and counterspeech, and helps to prevent the dataset from being dominated by simple samples.

In particular, we use 2 methods to develop the AugCOLD dataset: creating synthetic data through prompt-based model generation and crawling data from the real world by using detectors.

#### Prompt-based data augmentation

Generating synthetic data via large pretrained language models has been demonstrated to be an effective way for data augmentation.

In this work, we perform data augmentation by generating synthetic data with few-shot prompts on GLM-10B and GLM-large [5].

##### Prompt design

We construct various 2-shot prompts in particular to broaden and diversify the scope and variety of the augmented data. Prompts consist of seed samples with annotated labels from COLDataset [16] and CDialBias [17]. Two types of prompts are designed based on the following 2 strategies.

•Prompt with binary label constraint. Create prompts by selecting seed samples with the same label (offensive or not) at random. For example, 2 offensive samples that refer to different topics or different target groups. Such prompts steer the model to generate offensive content while retaining its potential to produce data on a wider range of topics and target groups. This is advantageous for expanding the data coverage.

•Prompt for triggering hard cases. Seed samples for augmenting hard cases are mainly picked from CDialBias. This dataset focuses on social bias and considers several attitudes including bias, neutral, and anti-bias. Among them, biased expressions are comparatively subtle in comparison to other offenses, such as insults. Neutral and anti-bias expressions are nonoffensive but are more likely to be misclassified as offensive than other safe expressions. Therefore, these data can be utilized as seed samples for augmenting hard cases.

##### Quality filtering

For the synthetic data generated by the language model, its quality is difficult to guarantee. Therefore, we use Perplexity (PPL) to control text fluency. PPL is usually used to evaluate the performance of language models. For the test sentences in fact real and correct, the model will assign a lower perplexity for them, which denotes that the model is not perplexed by them and understand them well. Therefore, we believe that if a relatively reliable model is used to score the generated text, its PPL value can reflect its fluency to a certain extent. However, recent work finds that very low PPL cannot represent very high quality [41]. It is because the repetition of words or phrases will sharply down the PPL value, while repetition often occurs in generated texts. In this way, we cautiously use the PPL metric to filter the unfluency generations and only keep the synthetic data with PPL values between 10 and 100. Some examples of prompts for data augmentation are shown in Table 6.

#### Selection from real-world data

Besides model generation, we collect real-world data to enlarge the diversity of AugCOLD dataset, mainly through the following 2 ways.

##### Data selection with detector

First, we select data from existing academic datasets: (a) SimplifyWeibo (https://github.com/SophonPlus/ChineseNlpCorpus/blob/master/datasets/simplifyweibo_4_moods/intro.ipynb), which contains around 360k posts crawled from Sina Weibo. They are emotionally tagged with 4 emotions of happiness, anger, disgust, and fear. (b) Weibi Sentiment corpus (https://github.com/SophonPlus/ChineseNlpCorpus/blob/master/datasets/weibo_senti_100k/intro.ipynb), which contains about 100k posts crawled from Sina Weibo. It is proposed for sentiment analysis and has approximately 50k positive comments and 50k negative comments. (c) Douban Movie Short Comments Dataset (https://www.kaggle.com/datasets/utmhikari/doubanmovieshortcomments), which includes over 2 million short comments and viewpoints on 28 movies from the Douban Movie website.

We take the above datasets as candidates and then score them with the classifier, COLDetector [16], to determine whether or not each sample is offensive. Each sample is assigned a score between 0 and 1, indicating the probability that the sentence is offensive. Following that, we relatively uniformly pick samples from each score interval, such as 0-0.1, 0.1-0.2, etc. These data with varying scores are added to the AugCOLD dataset, making the data more varied.

##### Data selection with keywords

According to prior research, the automatic detection of offensive content could be hindered by the presence of sensitive keywords [16]. This is due to the fact that sensitive words might exist in both offensive and nonoffensive samples, and even the most offensive sensitive terms have a high likelihood of appearing in safe samples, such as anti-bias statements. Nevertheless, because the majority of samples containing keywords in the training data are offensive, once the model detects sensitive words in the input, it tends to disregard other features and incorrectly predict the input as offensive. This results in a high recall score but a low level of precision for the offensive detector.

To alleviate this problem and further increase data coverage, we collect data by keyword-matching method. Specifically, we crawl a large amount of data from platforms like Weibo and Zhihu. Due to the low density of offensive-related data, we manually collected 2.6k blacklist terms covering keywords related to offenses such as abusive, discriminatory, pornographic, and intimidating. Then, we selected 96k candidate offensive samples with keyword matching and randomly selected 24k candidate safe samples.

•Examples of sensitive keywords: 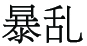 (riot), 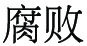 (corruption), 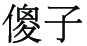 (stupidity), 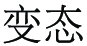 (perverts), 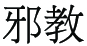 (cult), 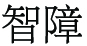 (mental retardation), 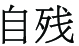 (self-harm), 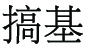 (faggotry)

#### AugCOLD dataset

We develop AugCOLD dataset, which includes 1,090k samples and is almost 29 times larger than the initial COLDataset. Detailed data statistics of AugCOLD dataset are presented in Table 7.

**Table 7.**
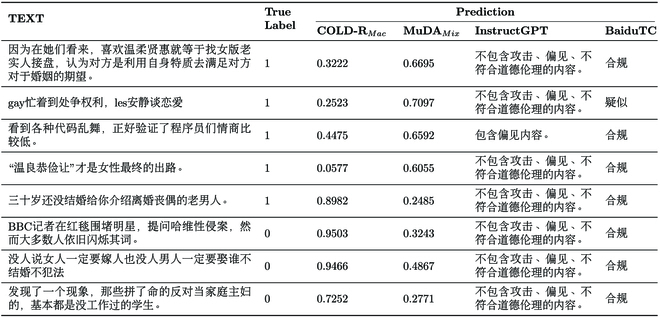
Case study on gathered HardTest. Each example has a binary “True Label”, with “1” denoting offensive content. This table includes the offensiveness probability assigned by COLD-R *_Mac_* and MuDA *_Mix_*, as well as the prediction from InstrucGPT and BaiduTC.

##### Lexical diversity

We investigate lexical diversity, where the number of unique unigrams in AugCOLD is double that of COLDataset (4.6k vs. 9.4k) and where the number of unique 5 g is about 33 times that of COLDataset (1,363k vs. 45,794k). This demonstrates that AugCOLD has a large increase in sample diversity and coverage. This is owed, in part, to the inclusion of real-world data, which brings the augmented dataset closer to the actual deployment scenario.

##### Offensiveness

**Fig. 2.**
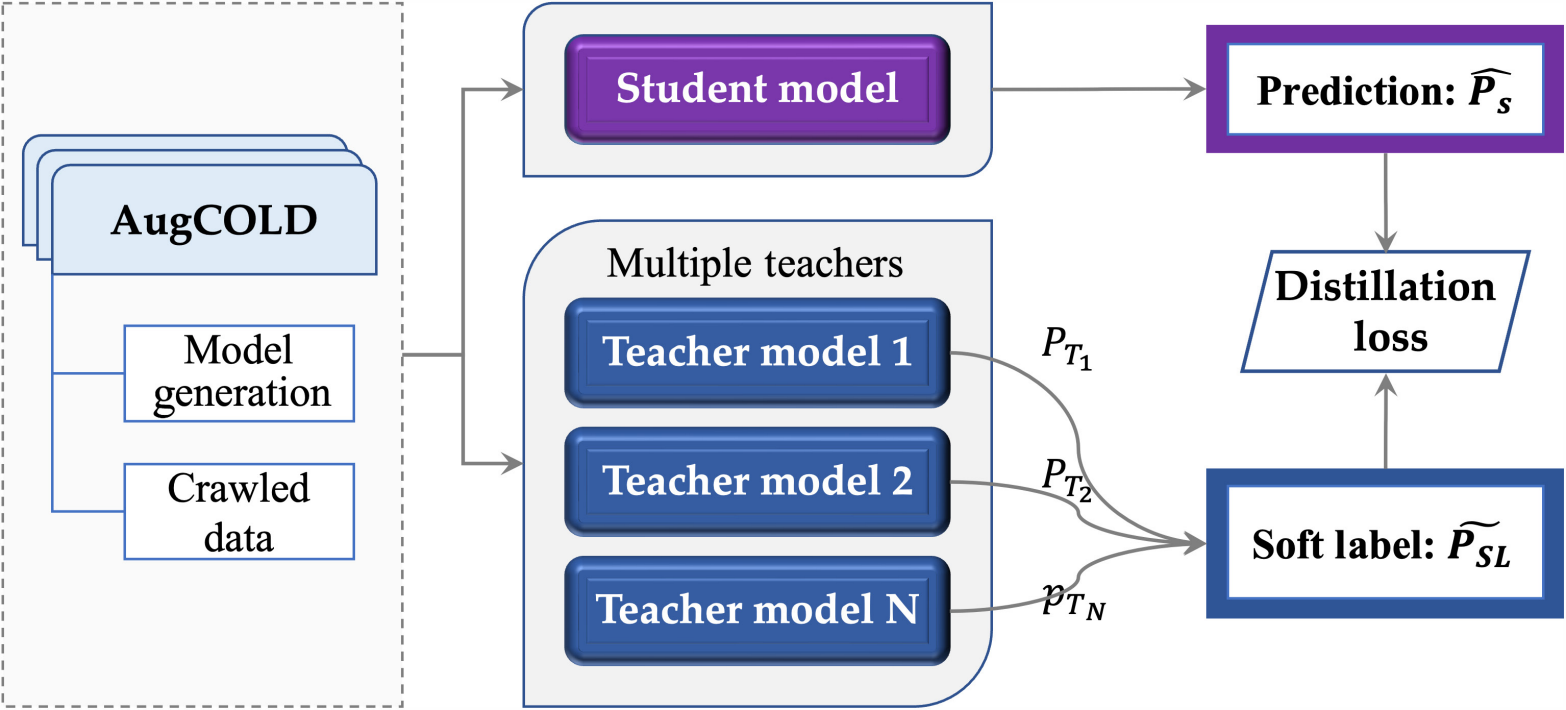
Multiteacher knowledge distillation framework.

To better explore the offensive distribution, we analyze the offensiveness of AugCOLD dataset. Utilizing *N* teacher models, we obtain multiple probability outputs (*P*_1_, *P*_2_, …*P_N_*) for each sample and then calculate the average (*AvgScore*) and maximum (*MaxScore*) offensive scores for each sample: $\text{AvgScore}=\frac{1}{N}\sum_{i=1}^{N}‍P_{i}$, *MaxScore* =  *max* (*P_i_*). We count the number of examples whose offensive score fell within each range, and the results are shown in Fig. 2. In general, samples with an average toxicity score (*AvgScore*) between 0.3 and 0.7 can be considered more challenging for the detector, and this portion of the data accounts for approximately 42%, showing that the simple sample will not overwhelm the dataset.

It can be observed that AugCOLD dataset can cover a wider range of offensive levels, hence satisfying the diversity requirement of offensiveness distribution.

##### Quality generated data

Due to the limitations of the language model’s generation capability, the augmented synthetic data may contain repetitions and grammatical faults. To verify the quality of augmented synthetic data, we randomly select 200 samples and manually evaluate their fluency. Of them, 185/200 (92.50%) are considered to be fluent and easily mistaken as human-written data. After PPL filtering, 191 samples remained, of which 183/191 (95.81%) are fluent. This reveals that PPL filtering may effectively exclude poor-quality samples and enhance the quality of the remaining data. Examples of augmented synthetic data are shown in Table 2.

### Multiteacher Knowledge Distillation Framework

**Fig. 3.**
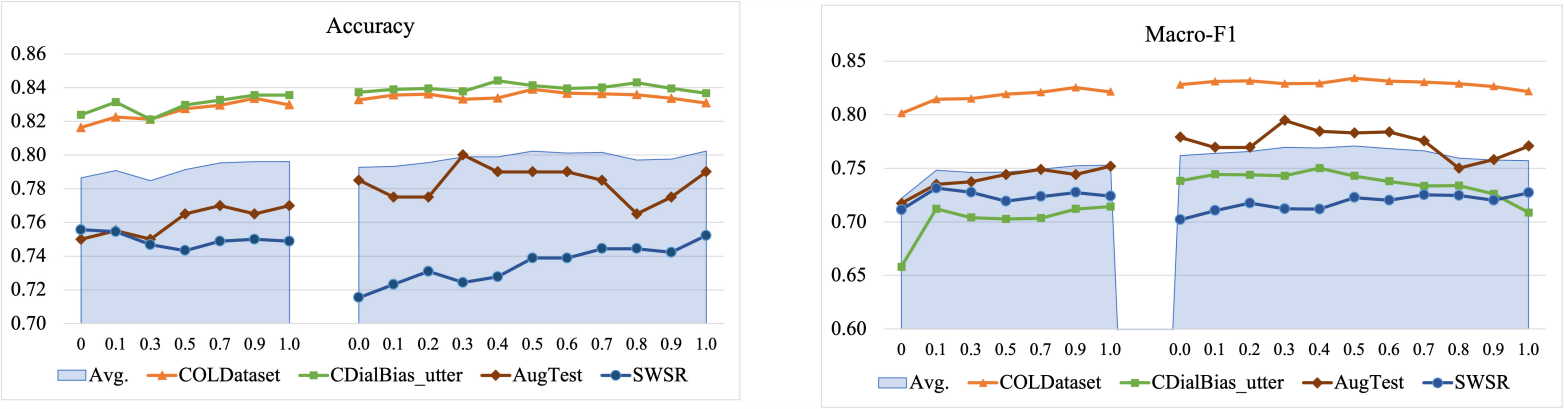
Accuracy and macro-f1 score with varying weights *γ* of soft labels considered in loss function. The results of 2-step knowledge distillation are shown: distillation on AugCOLD and continuing distillation on all supervised data.

Limited by the quality and quantity of training data, existing Chinese offensive detectors confront significant challenges in terms of generalization on new topics and robustness to hard cases when they are deployed. Recent studies have shown that unsupervised data with pseudo-labels can improve the performance of detectors. Motivated by this, we construct a large-scale unsupervised dataset AugCOLD and explore the application of Multiteacher Knowledge Distillation with the Augmented dataset (MuDA). With such a framework, as shown in Fig. 3, we can distill knowledge from both unsupervised data and multiple teachers to boost the performance of student model. To achieve the above goals, the construction of unsupervised datasets and the training of multiteacher networks are the 2 most important parts.

#### Construction of unsupervised dataset

Unsupervised data should be diversified and broad in scope. Yet, gathering such information is a significant undertaking. Because of the healthy communication environment in social networks, the diffusion of offensive samples in the actual world is highly limited. Second, available datasets are overburdened with simple examples, making it challenging for the compiled detector to deal with complex samples such as concealed toxicity and counterspeech. To address the difficulties stated above, we construct unsupervised data AugCOLD, which is an extension of COLDataset [16]. To maximize data coverage and diversity, we collect data from 2 sources: real-world data crawling and data augmentation with generation models.

It is important to highlight that during the data collection process of AugCOLD, we obtain raw labels that are automatically assigned based on the label constraints in prompt-based generation and the predictions from detector/keyword-based data selection. However, in our pilot experiments, we have identified inherent inaccuracies in these raw labels. This can be attributed to the limitations of the detectors or the possibility that the generated samples might not strictly adhere to the labeling instructions provided in the prompt. Therefore, we have made a decision to exclude these raw labels and rely solely on the augmented data generated by AugCOLD. The details of AugCOLD development are given in section AugCOLD Development.

#### Building the multiteacher network

The multiteacher network has multiple independent offensive detectors that are usually trained on various datasets, guaranteeing that they can successfully handle a variety of inputs, even hard cases, and then giving the student model strong robustness and generalization. Considering that the Chinese data is limited in quantity and scope, we employ both Chinese data and English translation data to train the teacher model in order to make it capable of handling a variety of input cases.

With the pretrained teacher models, unsupervised data can be scored and then soft labels are generated. These soft labels are served as a training signal and guide the training of the student model, thereby improving the detector’s robustness and generalization.

Specifically, in our distillation framework, *N* independent binary classification models served as teachers: *T*_1_, *T*_2_, …, *T_N_*. For each sample in training data, these teachers generate corresponding class prediction probabilities: *P*_1_, *P*_2_, …, *P_N_*. Then, each sample is assigned with soft label ${\tilde{P}}_{\mathrm{SL}}$ and pseudo hard label: *y_PH_*. The student model is trained by minimizing the distillation loss *L_DM_*:

$${\tilde{P}}_{\mathrm{SL}}=\sum_{i=1}^{N}‍w_{i}\cdot P_{i}$$ (1)

$$y_{\mathrm{PH}}=\left\{\begin{array}{cc} 1 & \text{if}\;{\tilde{p}}_{\mathrm{SL}}>=0.5\\ 0 & \;\text{if}\;{\tilde{p}}_{\mathrm{SL}}<0.5 \end{array}\right.$$ (2)

$$\begin{array}{c} L_{\mathrm{DM}}=\left(1-\gamma \right)\cdot \mathrm{CE}\left(y_{\mathrm{PH}},{\hat{y}}_{s}\right)+\gamma \cdot \mathrm{KL}\left({\tilde{P}}_{\mathrm{SL}}\left|\right|{\hat{y}}_{s}\right) \end{array}$$ (3)

in which ${\hat{y}}_{s}$ is the predicted probability of student model, *CE*(·) is cross-entropy loss, *KL*(·) is the Kullback–Leibler divergence loss, and *w_i_* and *γ* are the hyperparameters. In experiments, *w_i_* is set to 1/*N*.

## Data Availability

The datasets generated and analyzed during the current study are available from the corresponding author upon reasonable request.
